# Feasibility Study of Endoscopic Surgery for Spontaneous Intracerebral Hemorrhage with Large Hematoma: a Comparison with Craniotomy Using Propensity Score Matching Analysis

**DOI:** 10.1007/s12028-024-02085-0

**Published:** 2024-08-27

**Authors:** Min Cui, XiaoYong Tang, WeiMing Xiong, YongBing Deng, Qiang Yang

**Affiliations:** Department of Neurosurgery, Chongqing Emergency Medical Center, Chongqing University Central Hospital, Chongqing, China

**Keywords:** Intracerebral hemorrhage, Cerebral edema, Craniotomy, Decompressive craniectomy, Endoscopic surgery

## Abstract

**Background:**

Spontaneous intracerebral hemorrhage (ICH) with large hematomas is commonly treated with craniotomy combined with decompressive craniectomy, procedures that involve huge trauma and require subsequent cranioplasty. Recently, endoscopic surgery has shown significant promise in treating ICH, but its feasibility for large hematomas remains uncertain. Therefore, this study aims to compare endoscopic surgery with craniotomy and to evaluate the efficacy and safety of endoscopic surgery in treating large hematomas ICH.

**Methods:**

A retrospective analysis was conducted on the clinical data from patients with spontaneous supratentorial ICH and hematoma volumes exceeding 50 mL who underwent either endoscopic surgery or craniotomy. Propensity score matching analysis was employed to reduce selection bias. The efficacy and safety of endoscopic surgery were evaluated by analyzing blood loss, postoperative edema, mortality rate, complications, and the Glasgow Outcome Scale (GOS) at 6-month follow-up.

**Results:**

A total of 113 cases that met the criteria were collected, with 65 in the endoscopic surgery group and 48 in the craniotomy group. After propensity score matching, each group contained 34 cases. The mean hematoma volume was 64.84 ± 11.02 mL in the endoscopy group and 66.57 ± 12.77 mL in the craniotomy group (*p* = 0.554). Hematoma evacuation rates were 93.27% in the endoscopy group and 89.34% in the craniotomy group (*p* = 0.141). The endoscopy group exhibited lower blood loss, shorter surgical time, and reduced postoperative edema volume at 24 h compared to the craniotomy group. The rate of pulmonary infection was slightly lower in the endoscopy group compared to the craniotomy group (70.59% vs. 91.18%, *p* = 0.031), but there were no statistically significant differences in overall complications and mortality rate between the two groups. GOS scores were similar in both groups at the 6-month follow-up.

**Conclusions:**

Endoscopic surgery is safe and feasible for treating spontaneous supratentorial ICH with large hematomas, demonstrating efficacy similar to that of craniotomy with decompressive craniectomy.

## Introduction

Spontaneous intracerebral hemorrhage (ICH) is a common subtype of stroke associated with high disability and mortality rates, imposing a substantial burden on families and society [1]. The mortality rate is particularly elevated for patients with large hematomas. Although current clinical research suggests limited benefits of surgical treatment for patients with ICH, surgical intervention is widely considered an effective lifesaving measure and is extensively employed in clinical practice [2, 3]. Craniotomy with or without decompressive craniectomy is a conventional surgical approach that effectively evacuate hematomas, alleviates mass effect, and reduces intracranial pressure (ICP), thereby offering a chance for patient survival. However, craniotomy is associated with huge trauma, a high incidence of complications, and the need for subsequent cranioplasty [4].

Endoscopy has been widely used in the treatment of ICH, with studies indicating superior outcomes compared to craniotomy for small to moderate hematomas [5–9]. However, research on the use of endoscopy for treating large hematomas is insufficient. Therefore, this study aims to compare endoscopic surgery and craniotomy for the treatment of large hematomas ICH to evaluate the feasibility and safety of endoscopic surgery.

## Methods

### Patient Data

A retrospective analysis was conducted on data from patients with spontaneous ICH admitted to the Chongqing Emergency Medical Center from January 2019 to June 2023. Prior to surgery, all patients or family members signed a surgical consent form. This study was approved by the hospital’s ethics committee.

All patients underwent computer tomography (CT) angiography to exclude conditions such as arteriovenous malformations and aneurysms. Inclusion criteria for the study were the following: (1) spontaneous supratentorial ICH, (2) hematoma volume larger than 50 mL or evidence of unilateral cerebral herniation based on CT or clinical presentation, (3) Glasgow Coma Scale (GCS) score of 4 or higher, (4) admission within 24 h of onset, and (5) age over 18 years. Exclusion criteria were the following: (1) hemorrhage caused by trauma, tumors, aneurysms, or cerebrovascular malformations; (2) coagulation disorders; (3) multiple ICHs; (4) significant organ failure, such as cardiac or pulmonary dysfunction; and (5) history of stroke.

On admission, all patients received tailored symptomatic treatment, including hemostasis, blood pressure and blood glucose control, dehydration, and nutritional support. The choice of surgical approach was based on various factors, including the patient’s condition, the surgeon’s expertise in the surgical technique, and the preferences of the patient’s family.

### Surgical Treatment

#### Endoscopic Surgery

**Fig. 1:**
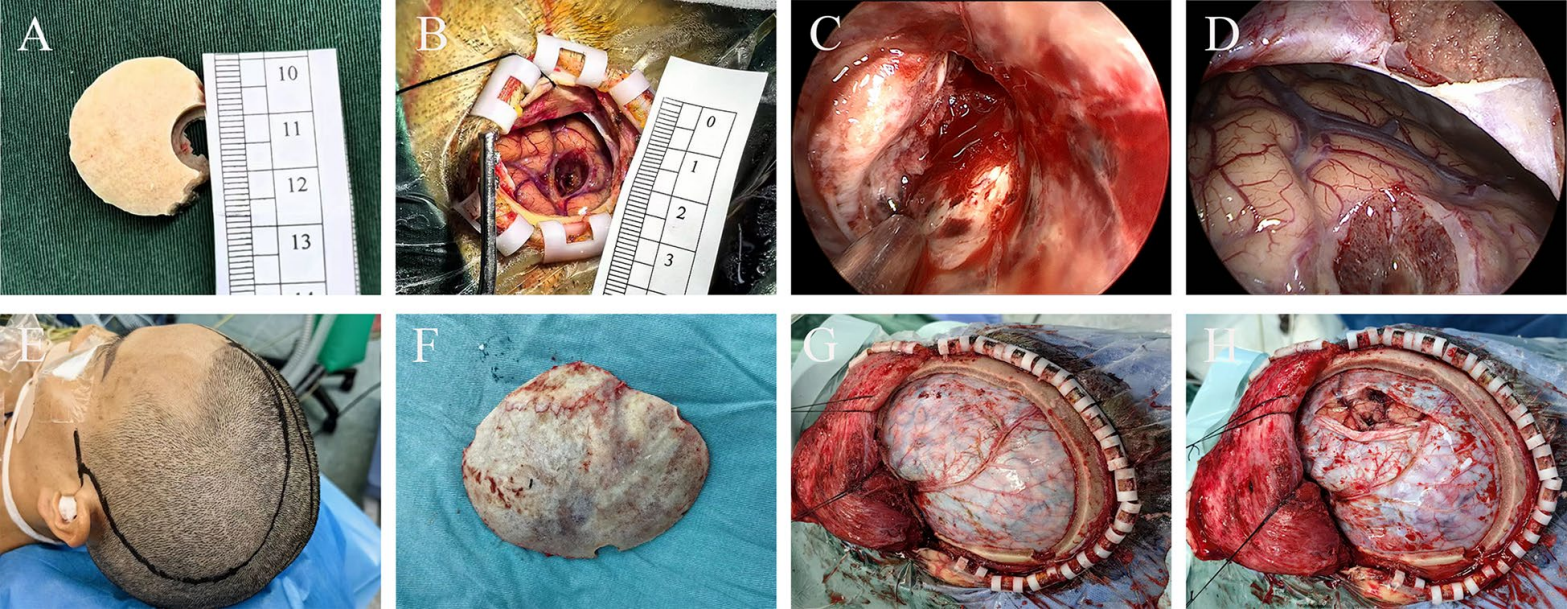
Illustration of intraoperative situations during endoscopic and craniotomy surgeries. **a–d**, Depiction of the endoscopic surgery. **a**, Size of the bone flap. **b**, Size of the skin flap and cortical incision. **c**, The hematoma cavity after evacuation. **d**, Collapse of brain tissue post evacuation. **e–h**, Representation of the craniotomy surgery. **e**, Incision line for craniotomy. **f**, Size of the bone flap removed. **g**, Condition of the surgical area before hematoma evacuation. **h**, Condition of the surgical area after hematoma evacuation

**Fig. 2:**
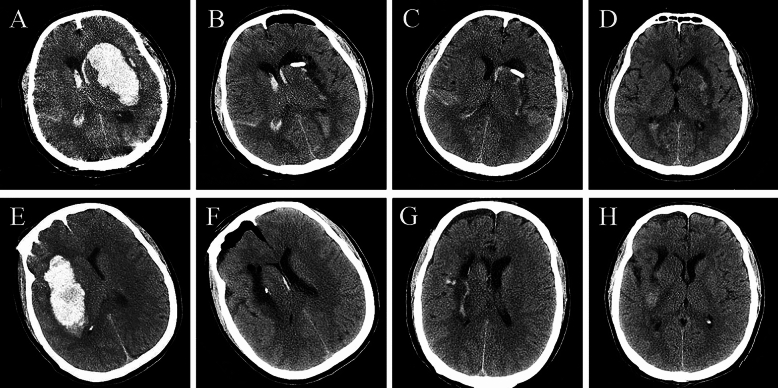
Illustration of endoscopic surgery. **a**, Preoperative CT image showing a large hematoma in the left hemisphere. **b–d**, Sequential follow-up CT images at 24 h, 3 days, and 7 days post operation displaying nearly complete evacuation of the hematoma, minimal postoperative edema, and reduced mass effect. **e**, Preoperative head CT image of another patient. **f–h**, CT images at 24 h, 3 days, and 9 days post operation. CT computer tomography

Endoscopic surgery was conducted under general anesthesia using endoscopy. The surgical approach was determined preoperatively based on the long axis of the hematoma as seen on CT scans. The procedure involved a linear incision, creation of a small bone flap, and insertion of a transparent plastic dilator following a cruciate durotomy. Hematoma evacuation and hemostasis were achieved using a rigid neuroendoscope. The bone flap was replaced after evacuation (Figs. 1a–d and 2).

#### Craniotomy

**Fig. 3:**
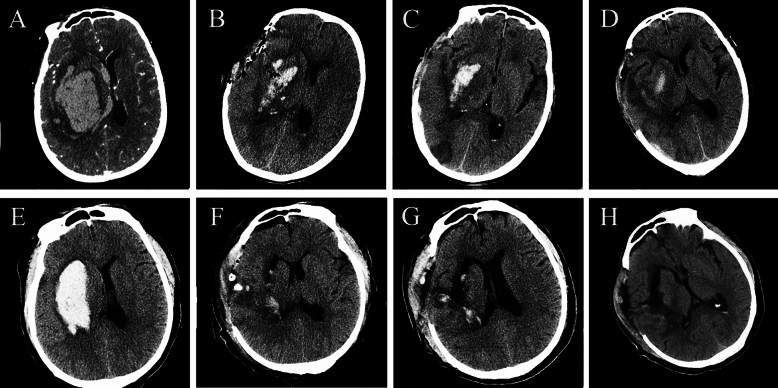
Illustration of craniotomy with decompressive craniectomy. **a**, Preoperative head CT image showing a large hematoma in the right hemisphere. **b–d**, CT images at 24 h, 3 days, and 10 days post operation displaying significant hematoma evacuation with some postoperative edema in the surgical area. **e**, Preoperative head CT image of another patient. **f–h**, CT images at 24 h, 3 days, and 9 days post operation. CT computer tomography

Craniotomy surgery was performed under general anesthesia following a preoperative design of the surgical incision based on CT scans. The procedure involved scalp incision, cranial drilling, and bone flap creation, followed by opening the dura mater and incising the cortex to access the hematoma cavity without disrupting nerves and blood vessels. Hematoma evacuation was conducted under direct vision or with the assistance of a microscope to ensure hemostasis. Decompression was achieved by suturing the dura mater without replacing the bone flap, followed by scalp closure to conclude the surgery (Figs. 1e–h and 3).

Drainage tubes were routinely placed in the hematoma cavity or lateral ventricle, and an ICP probe was placed in the ventricle or subcortical area at the end of the surgery for continuously monitoring for 5–7 days postoperatively. All patients were then transferred to the neurological intensive care unit for critical care management. Head CT scans were performed after surgery and at 24 h to assess hematoma clearance and detect any rebleeding. Subsequent CT scans were conducted based on the patient’s condition. Symptomatic treatment included osmotic diuretics, such as mannitol, glycerol fructose, furosemide, and albumin. In some cases, contralateral ventricular drainage was performed postoperatively to control ICP. Decompressive craniectomy involving the removal of a bone flap approximately 10 × 12 cm in size was considered for cases in which ICP remained elevated. Tracheostomy was performed in cases of severe impairment of consciousness, severe pulmonary infection, or poor sputum expectoration to maintain airway patency.

Patient information, including sex, age, blood pressure at admission, history of hypertension, history of diabetes, coagulation function (international normalized ratio), intraventricular hemorrhage, cerebral herniation, GCS score, and time from onset to surgery, was recorded. Surgical data collected included duration of surgery, blood loss, preoperative and postoperative hematoma volume, hematoma evacuation rate, 24-h postoperative edema volume, ICP, rebleeding, complications (such as cerebral infarction, intracranial infection, pulmonary infection, gastrointestinal bleeding, epilepsy, and deep vein thrombosis), mortality rate, and length of hospital stay. Glasgow Outcome Scale (GOS) scores was recorded at 6 months.

The 24-h postoperative edema volume was used as an indicator of iatrogenic injury during surgery [7], with hematoma and edema volumes measured using 3D Slicer. The hematoma evacuation rate was calculated using the following formula: (preoperative hematoma volume–postoperative hematoma volume)/preoperative hematoma volume × 100%. GOS scores were interpreted as follows: 1 for death, 2 for vegetative survival, 3 for severe disability, 4 for moderate disability, and 5 for independent living. A score of 4–5 indicated a good recovery [10].

#### Statistical Analysis

To mitigate selection bias and other confounding factors, a 1:1 propensity score matching analysis (caliper 0.2) was performed based on the following baseline characteristics: hematoma volume, ventricular hemorrhage, GCS score, hematoma location, and the time to surgery. Continuous variables with a normal distribution were presented as mean ± standard deviation and analyzed using the *t*-test. Nonnormally distributed variables were presented as the medians with interquartile ranges and analyzed using the Mann–Whitney *U*-test. Categorical data were represented as numbers and percentages and analyzed using the *χ*^2^ test. A* p* value less than 0.05 was considered statistically significant.

## Results

### Baseline Data

The study analyzed 113 cases that met the inclusion criteria, with 65 cases in the endoscopic surgery group and 48 cases in the craniotomy group. Using propensity score matching at a 1:1 ratio, 34 cases were selected for each group. Post–propensity score matching analysis revealed no statistically significant differences in baseline characteristics between the endoscopic surgery and craniotomy groups. The mean age was 53.38 ± 11.36 years in the endoscopy group and 53.79 ± 11.65 years in the craniotomy group (*p* = 0.883). Both groups had 67.65% male patients. In the endoscopy group, 15 cases (44.12%) had a GCS score of 4–8, compared to 11 cases (32.35%) in the craniotomy group (*p* = 0.318). Hemorrhage in the basal ganglia region was present in most cases in both groups (88.24% vs. 91.18%, *p* = 0.690). The mean preoperative hematoma volume was 64.84 ± 11.02 mL in the endoscopy group and 66.57 ± 12.77 mL in the craniotomy group (*p* = 0.554). In the endoscopy group, 9 cases (26.47%) exhibited unilateral cerebral herniation, compared to 11 cases (32.35%) in the craniotomy group (*p* = 0.595). There were no statistically significant differences in admission blood pressure, coagulation function (international normalized ratio), creatinine, history of hypertension, history of diabetes, presence of intraventricular hemorrhage, or time from onset to surgery between the two groups (Table 1).

**Table 1:** Baseline characteristics of two groups after propensity score matching

	Endoscopy group (*n* = 34)	Craniotomy group (*n* = 34)	*p* value
Age, mean ± SD, years	53.38 ± 11.36	53.79 ± 11.65	0.883
Male, *n* (%)	23 (67.65)	23 (67.65)	1.000
SBP, mean ± SD, mm Hg	178.50 ± 25.30	179.03 ± 29.32	0.937
Platelet count, median (IQR), × 10^9^/L	195.00 (162.25–256.00)	231.50 (167.50–233.75)	0.394
INR, mean ± SD	1.02 ± 0.085	1.00 ± 0.115	0.519
Creatinine, median (IQR), μmol/L	61.55 (47.08–77.13)	64.20 (51.75–78.25)	0.646
Hypertension, *n* (%)	23 (67.65)	23 (67.65)	1.000
Diabetes, *n* (%)	2 (5.88)	2 (5.88)	1.000
IVH, *n* (%)	19 (55.88)	17 (50.00)	0.627
Pre-GCS, *n* (%)			0.318
4–8	15 (44.12)	11 (32.35)	
≥ 9	19 (55.88)	23 (67.65)	
Time from onset to surgery, median (IQR), hours	9.00 (6.00–13.00)	7.50 (5.75–12.25)	0.389
Pre-ICH volume, mean ± SD, mL	64.84 ± 11.02	66.57 ± 12.77	0.554
Cerebral herniation, *n* (%)	9 (26.47)	11 (32.35)	0.595
Hematoma location, *n* (%)			0.690
Basal ganglia	30 (88.24)	31 (91.18)	
Lobe	4 (11.76)	3 (8.82)	

*GCS* Glasgow Coma Scale, *ICH* Intracerebral hemorrhage, *INR* International normalized ratio, *IQR* Interquartile range, *IVH* intraventricular hemorrhage, *SBP* systolic blood pressure

### Surgical and Prognostic Evaluation

Both endoscopic surgery and craniotomy effectively evacuate the hematomas, with evacuation rates of 93.27% and 89.34%, respectively, showing no statistical difference (*p* = 0.141). However, blood loss during endoscopic surgery was significantly lower at 50 mL compared to 450 mL in the craniotomy group (*p* < 0.001). The average surgical time for endoscopic surgery was also significantly shorter than for craniotomy (140 vs. 205 min, *p* < 0.001). Additionally, the average 24-h postoperative edema volume was significantly lower in the endoscopy group at 28.49 mL, compared to 61.85 mL in the craniotomy group (*p* < 0.001). There was no significant difference in postoperative 24-h ICP between the two groups (11.58 ± 5.10 mm Hg vs. 11.79 ± 4.46 mm Hg, *p* = 0.858) (Table 2).

**Table 2:** Comparison of efficacy and outcome between two groups

	Endoscopy group (*n* = 34)	Craniotomy group (*n* = 34)	*p* value
Postoperative hematoma, median (IQR), mL	4.04 (1.96–10.15)	6.50 (2.61–10.50)	0.128
Evacuation rate, median (IQR), %	93.27 (84.89–97.19)	89.34 (83.37–95.78)	0.141
Blood loss, median (IQR), mL	50 (50–100)	450 (275–600)	< 0.001
Duration of surgery, median (IQR), min	140 (90–205)	205 (180–300)	< 0.001
Brain edema, median (IQR), mL	28.49 (19.77–34.10)	61.85 (39.81–83.13)	< 0.001
ICP, mean ± SD, mm Hg	11.58 ± 5.10	11.79 ± 4.46	0.858
Rebleeding, *n* (%)	4 (11.76)	4 (11.76)	1.000
Mortality rate, *n* (%)	2 (5.88)	3 (8.82)	0.642
Hospital stay, median (IQR), days	32.50 (25.75–39.00)	48.00 (31.00–67.50)	0.002
GOS score, *n* (%)			0.798
1–3	23 (67.65)	22 (64.71)	
4–5	11 (32.35)	12 (35.29)	

*GOS* Glasgow Outcome Scale, *ICP* Intracranial pressure, *IQR* Interquartile range

In both the endoscopic and craniotomy groups, four patients (11.76%) experienced postoperative rebleeding, all of which were managed with conservative treatment, avoiding the need for a second surgery. Regarding postoperative complications, the endoscopy group had 1 case (2.94%) of cerebral infarction, 4 cases (11.76%) of intracranial infection, 24 cases (70.59%) of pulmonary infection, 7 cases (20.59%) of gastrointestinal bleeding, 1 case (2.94%) of epilepsy, and 3 cases (8.82%) of deep vein thrombosis. In the craniotomy group, there were 4 cases (11.76%) of cerebral infarction, 3 cases (8.82%) of intracranial infection, 31 cases (91.18%) of pulmonary infection, 10 cases (29.41%) of gastrointestinal bleeding, 4 cases (11.76%) of epilepsy, and 7 cases (20.59%) of deep vein thrombosis. Although the rate of pulmonary infection was slightly lower in the endoscopy group, there were no statistically significant differences in overall complications between the two groups (Table 3).

**Table 3:** Comparison of postoperative complication between two groups

	Endoscopy group (*n* = 34)	Craniotomy group (*n* = 34)	*p* value
Postoperative complication, *n* (%)	27 (79.41)	31 (91.18)	0.171
Cerebral infarction, *n* (%)	1 (2.94)	4 (11.76)	0.353
Intracranial infection, *n* (%)	4 (11.76)	3 (8.82)	1.000
Pulmonary infection, *n* (%)	24 (70.59)	31 (91.18)	0.031
Gastrointestinal bleeding, *n* (%)	7 (20.59)	10 (29.41)	0.401
Epilepsy, *n* (%)	1 (2.94)	4 (11.76)	0.353
Thrombus, *n* (%)	3 (8.82)	7 (20.59)	0.171

The endoscopy group had two patients (5.88%) who died, whereas the craniotomy group had three patients (8.82%), with no significant difference (*p* = 0.642). The length of hospital stay was significantly shorter for the endoscopy group, averaging 32.50 days compared to 48.00 days for the craniotomy group (*p* = 0.002). At the 6-month follow-up, 11 cases (32.35%) in the endoscopy group achieved a favorable outcome (GOS score of 4–5), which was comparable to the craniotomy group with 12 cases (35.29%) (*p* = 0.798) (Table 2).

## Discussion

The debate over the surgical treatment of ICH remains unresolved, with no current consensus. Surgery theoretically offers several benefits, including hematoma evacuation, alleviation of mass effect, relief of neurological compression symptoms, improved perfusion, and reduction of secondary injury. These advantages have made surgery a common clinical practice [11–13]. However, large clinical studies such as Surgical Trial in Intracerebral Hemorrhage (STICH) and the STICH II have not demonstrated significant benefits of surgical treatment for patients with ICH [2, 3]. Notably, these studies excluded some critically ill cases. However, surgery could potentially save lives and improve prognoses in this subgroup. Currently, there is a scarcity of studies specifically analyzing surgical treatments, particularly endoscopic surgery, in critically ill patients with ICH with large hematomas. Most existing research consists of subgroup analyses rather than comprehensive studies.

For patients with large hematomas, the mass effect and associated brain edema can elevate ICP, potentially leading to cerebral herniation and posing a life-threatening risk. The prevailing surgical approach is craniotomy for hematoma evacuation with or without decompressive craniectomy. Craniotomy effectively evacuates the hematoma, achieves hemostasis under direct vision, and reduces mortality. Decompressive craniectomy can further lower ICP following bone flap removal, significantly reduce midline shift, and provide a buffer for rebleeding and delayed edema [14]. Research indicates that combining decompressive craniectomy with craniotomy yields better therapeutic outcomes than craniotomy alone [15]. However, craniotomy involves excessive mechanical traction, electrocoagulation, and cauterization, which can cause huge brain injury. Additionally, the procedure is lengthy and associated with many postoperative complications. Craniectomy also alters the normal cranial structure, potentially leading to unpredictable side effects, such as subdural fluid accumulation and hydrocephalus, which may require secondary cranioplasty. These factors increase patient trauma and associated risks, rendering craniectomy a less preferred option [16–18].

In the context of adequate hematoma evacuation, edema is a crucial factor influencing ICP following surgical treatment. Edema primarily originates from two sources: the hematoma and its metabolic products and the impact of surgery on the brain. Posthemorrhagic edema mainly results from a cascade reaction, including the inflammatory response and oxidative stress, which leads to the development of edema that progressively worsens over time [19–21]. Therefore, early surgery can alleviate the edema caused by the hematoma itself and mitigate secondary damage, which is critical for surgical success. However, iatrogenic injuries, such as mechanical traction, electrocoagulation, and cauterization, can exacerbate edema. Postoperative edema typically peaks 24 h after surgery and becomes more pronounced with greater surgical trauma [7].

In theory, early minimally invasive surgery can minimize brain edema, potentially eliminating the need for decompressive craniectomy. Minimally invasive procedures, including puncture drainage and endoscopic surgery, cause minimal damage, particularly endoscopic surgery, which is widely used in clinical treatment because of its excellent visualization [22, 23]. Endoscopic surgery causes minimal injury, reducing interference and damage to the brain tissue surrounding the hematoma, thereby reducing iatrogenic edema. This reduction in edema is crucial for managing ICP post operation. Endoscopic surgery provides a clear, multiangle operative field, allowing for comprehensive observation of pathological tissues and bleeding sites. This leads to more effective hematoma evacuation and hemostasis, resulting in a high hematoma evacuation rate and a low rebleeding rate. Multiple studies have confirmed that for small and medium-sized ICHs, endoscopic surgery reduces duration of surgery, decreases surgical complications, and improves prognosis, surpassing craniotomy [7, 24, 25]. For large hematoma ICHs, early endoscopic surgery can clear most of the hematoma, minimize injury and edema, mitigate mass effect, and maintain lower ICP, potentially eliminating the need for decompressive craniectomy. Recent research supports the feasibility and safety of this approach. Ye et al. [26] conducted a retrospective analysis of 112 patients with large hematoma ICHs, finding no significant differences in mortality rates and modified Rankin Scale scores between the craniotomy group and the endoscopy group. However, the endoscopy group experienced fewer postoperative complications compared to the craniotomy group [26].

Leveraging our center’s neuroendoscopy expertise, we have applied endoscopic surgery in various types of ICH and traumatic brain injury [27]. In ICH surgeries, we use a miniaturized endoscopic channel (outer diameter 1.1 cm, inner diameter 0.9 cm) to minimize brain injury. During localization, we carefully select the direction along the long axis of the hematoma to protect neural fiber bundles as much as possible. To minimize interference with the hematoma–brain tissue interface and reduce delayed edema, we operate strictly within the hematoma cavity during evacuation. For oozing of blood, hemostasis is achieved using compression with hemostatic gauze. In cases of vascular bleeding, the vessel is lifted before electrocoagulation and cauterization to minimize the impact on the brain. The key to successful surgery is ensuring sufficient hematoma evacuation to mitigate the mass effect of residual hematoma and the edema generated during its resolution. The endoscope’s excellent illumination enables clear observation of each bleeding point. While ensuring hemostasis, it is crucial to avoid overfilling the hematoma cavity with excessive hemostatic material. After hematoma clearance, brain pulsation and collapse should be carefully observed. If brain pulsation is good and the collapse depth is more than 0.5–1.0 cm below the bone window, the bone flap can be replaced.

In our study, endoscopic surgery demonstrated comparable hematoma evacuation rates to craniotomy, while significantly reducing surgical times and blood loss. Analysis of the 24-h postoperative CT scans revealed a significantly lower volume of edema in the endoscopic surgery group compared to the craniotomy group, indicating that iatrogenic trauma from endoscopic surgery was notably less than that from craniotomy. After evacuating the majority of the hematoma and replacing the bone flap, ICP remained within normal ranges post endoscopic surgery, comparable to that observed in craniotomy, despite the increased cranial cavity volume resulting from decompressive craniectomy. Rebleeding rates were similar in both groups, with no need for second surgical treatments. The endoscopic surgery group exhibited a lower rate of pulmonary infection compared to the craniotomy group, likely attributed to reduced injury from endoscopic surgery, facilitating early postoperative ambulation. However, overall complications did not significantly differ between the two groups. Patients undergoing endoscopic surgery experienced faster recovery and shorter hospital stays than those in the craniotomy group. At the 6-month follow-up, there were no significant differences in the GOS scores between the two groups, indicating that endoscopic surgery did not provide obvious advantages in improving prognosis. This lack of distinction may be attributed to the severe primary neurological damage in patients with large hematoma ICH, indicating that even with minimally invasive surgery, there is no significant improvement in neurological function. However, endoscopic surgery has no significant impact on cosmetic appearance and eliminates the necessity for secondary cranioplasty, along with its associated trauma and risks.

Based on our experience, several critical factors must be considered in endoscopic surgery without craniectomy:

1. Time from onset to surgery: Early intervention can mitigate brain edema caused by the hematoma. Beyond 24 h, the edema becomes significant, and bone flap retention may lead to unmanageably high ICP postoperatively. However, research also indicates that ultra-early surgery (less than 6 h) may increase the risk of rebleeding, underscoring the importance of timing for surgery [28].
2. Age: Brain atrophy worsens with advancing age, increasing the compensatory ability for high ICP.
3. Location of the hematoma: The farther away from the brainstem, the lower the risk of cerebral herniation.
4. ICP monitoring: Accurate and dynamic ICP monitoring facilitates timely detection of severe risks, such as rebleeding and severe edema.
5. External ventricular drainage: This involves placing a ventricular drain on the same side during surgery or performing contralateral ventricular drainage postoperatively. It allows dynamic and precise control of ICP by draining cerebrospinal fluid and can be sustained for several days to manage ICP during the peak period of cerebral edema. Ventricular drainage carries minimal trauma risks, primarily bleeding during puncture and postoperative infection, with a low occurrence rate indicated by current research [29].
6. Intensive care unit (ICU): ICU care is often necessary for timely observation of potential changes in the patient’s condition. Multimodal monitoring and respiratory assistance equipment in the ICU can promptly detect and manage changes. In cases of severe pulmonary infection or airway obstruction, early tracheostomy should be considered.

Limitations of our study include its single-center and retrospective nature, as well as a relatively small number of cases. Endoscopic surgery demands a high level of skill, and the procedure’s success is closely linked to the surgeon’s proficiency, limiting the generalizability of our findings. It is crucial to consider comprehensively, as endoscopic surgery may not be suitable for all cases of large hematoma ICH treatment.

## Conclusions

For large spontaneous supratentorial ICHs, endoscopic surgery appears safe and feasible, with efficacy comparable to that of craniotomy with decompressive craniectomy. However, these findings warrant further validation through multicenter prospective randomized controlled trials.

## Data Availability

The data sets used and/or analyzed during the current study are available from the corresponding author on reasonable request.
